# Optical Coherence Tomography-Derived Changes in Plaque Structural Stress Over the Cardiac Cycle: A New Method for Plaque Biomechanical Assessment

**DOI:** 10.3389/fcvm.2021.715995

**Published:** 2021-11-04

**Authors:** Jiayue Huang, Fan Yang, Juan Luis Gutiérrez-Chico, Tianxiao Xu, Jigang Wu, Liang Wang, Rui Lv, Yan Lai, Xuebo Liu, Yoshinobu Onuma, Dalin Tang, Patrick W. Serruys, William Wijns, Shengxian Tu

**Affiliations:** ^1^School of Biomedical Engineering, Biomedical Instrument Institute, Shanghai Jiao Tong University, Shanghai, China; ^2^The Lambe Institute for Translational Medicine and Curam, National University of Ireland Galway, Galway, Ireland; ^3^Cardiology Department, Ruijin Hospital, Shanghai Jiao Tong University School of Medicine, Shanghai, China; ^4^University of Michigan-Shanghai Jiao Tong University Joint Institute, Shanghai Jiao Tong University, Shanghai, China; ^5^School of Biological Science and Medical Engineering, Southeast University, Nanjing, China; ^6^Department of Cardiology, Tongji Hospital, Tongji University School of Medicine, Shanghai, China; ^7^Mathematical Sciences Department, Worcester Polytechnic Institute, Worcester, MA, United States

**Keywords:** biomechanical assessment, finite element analysis, optical coherence tomography, plaque structural stress, plaque rupture

## Abstract

**Introduction:** Cyclic plaque structural stress has been hypothesized as a mechanism for plaque fatigue and eventually plaque rupture. A novel approach to derive cyclic plaque stress *in vivo* from optical coherence tomography (OCT) is hereby developed.

**Materials and Methods:** All intermediate lesions from a previous OCT study were enrolled. OCT cross-sections at representative positions within each lesion were selected for plaque stress analysis. Detailed plaque morphology, including plaque composition, lumen and internal elastic lamina contours, were automatically delineated. OCT-derived vessel and plaque morphology were included in a 2-dimensional finite element analysis, loaded with patient-specific intracoronary pressure tracing data, to calculate the changes in plaque structural stress (ΔPSS) on vessel wall over the cardiac cycle.

**Results:** A total of 50 lesions from 41 vessels were analyzed. A significant ΔPSS gradient was observed across the plaque, being maximal at the proximal shoulder (45.7 [32.3, 78.6] kPa), intermediate at minimal lumen area (MLA) (39.0 [30.8, 69.1] kPa) and minimal at the distal shoulder (35.1 [28.2, 72.3] kPa; *p* = 0.046). The presence of lipidic plaques were observed in 82% of the diseased segments. Larger relative lumen deformation and ΔPSS were observed in diseased segments, compared with normal segments (percent diameter change: 8.2 ± 4.2% vs. 6.3 ± 2.3%, *p* = 0.04; ΔPSS: 59.3 ± 48.2 kPa vs. 27.5 ± 8.2 kPa, *p* < 0.001). ΔPSS was positively correlated with plaque burden (*r* = 0.37, *p* < 0.001) and negatively correlated with fibrous cap thickness (*r* = −0.25, *p* = 0.004).

**Conclusions:** ΔPSS provides a feasible method for assessing plaque biomechanics *in vivo* from OCT images, consistent with previous biomechanical and clinical studies based on different methodologies. Larger ΔPSS at proximal shoulder and MLA indicates the critical sites for future biomechanical assessment.

## Introduction

Spontaneous plaque rupture and subsequent thrombosis are recognized as the leading pathogenic mechanism for acute coronary syndrome (ACS), one of the major causes of mortality worldwide (1–3). Thin cap fibroatheroma (TCFA) has been postulated as the phenotype responsible for plaque rupture (3–8). However, limited specificity was observed for TCFA in predicting future coronary events, urging the need to further define meaningful surrogates for rupture-prone plaque identification (9–12).

From a biomechanical point of view, long-term repetitive superficial stress, generated by the pulsatile coronary pressure wave, might weaken the fibrous cap and ultimately lead to its fatigue and rupture (13, 14). Thus, the evaluation of cyclic plaque structural stress might add prognostic value for future cardiac events and subsequently for ACS prevention. Although direct *in vivo* measurement of plaque structural stress is not currently feasible, finite element analysis (FEA) might provide a reliable estimation (15). The prerequisites for accurate FEA are precise plaque morphology and composition, known mechanical properties of the different materials and precise model loads. Optical coherence tomography (OCT) provides optimal image resolution, enabling detailed visualization and precise characterization of plaque composition (16). Meanwhile, intracoronary pressure tracing from pressure wire could serve as an accurate load for cyclic plaque stress evaluation using FEA. The aim of this study was to propose a novel method to derive the changes in plaque structural stress during the cardiac cycle *in vivo* using a combination of OCT images and intracoronary pressure recordings.

## Materials and Methods

All patients from a previous prospective optical flow ratio (OFR) study with both OCT and fractional flow reserve (FFR) interrogation were screened for *post-hoc* analysis (17). Inclusion criteria were intermediate coronary lesions, defined as a diameter stenosis 40–90% by visual estimation. Exclusion criteria for FEA were: 1) bifurcation lesions with a side-branch ≥2mm; 2) diffuse coronary disease in the target vessel; 3) stented lesions; 4) intracoronary thrombus; 5) guidewire artifact in the OCT images; 6) incomplete intracoronary pressure recording, with baseline aortic pressure or distal coronary pressure at rest missing. Detailed description of the OCT acquisition and intracoronary pressure measurement has been previously reported (17).

### Representative Position Selection

**Figure 1:**
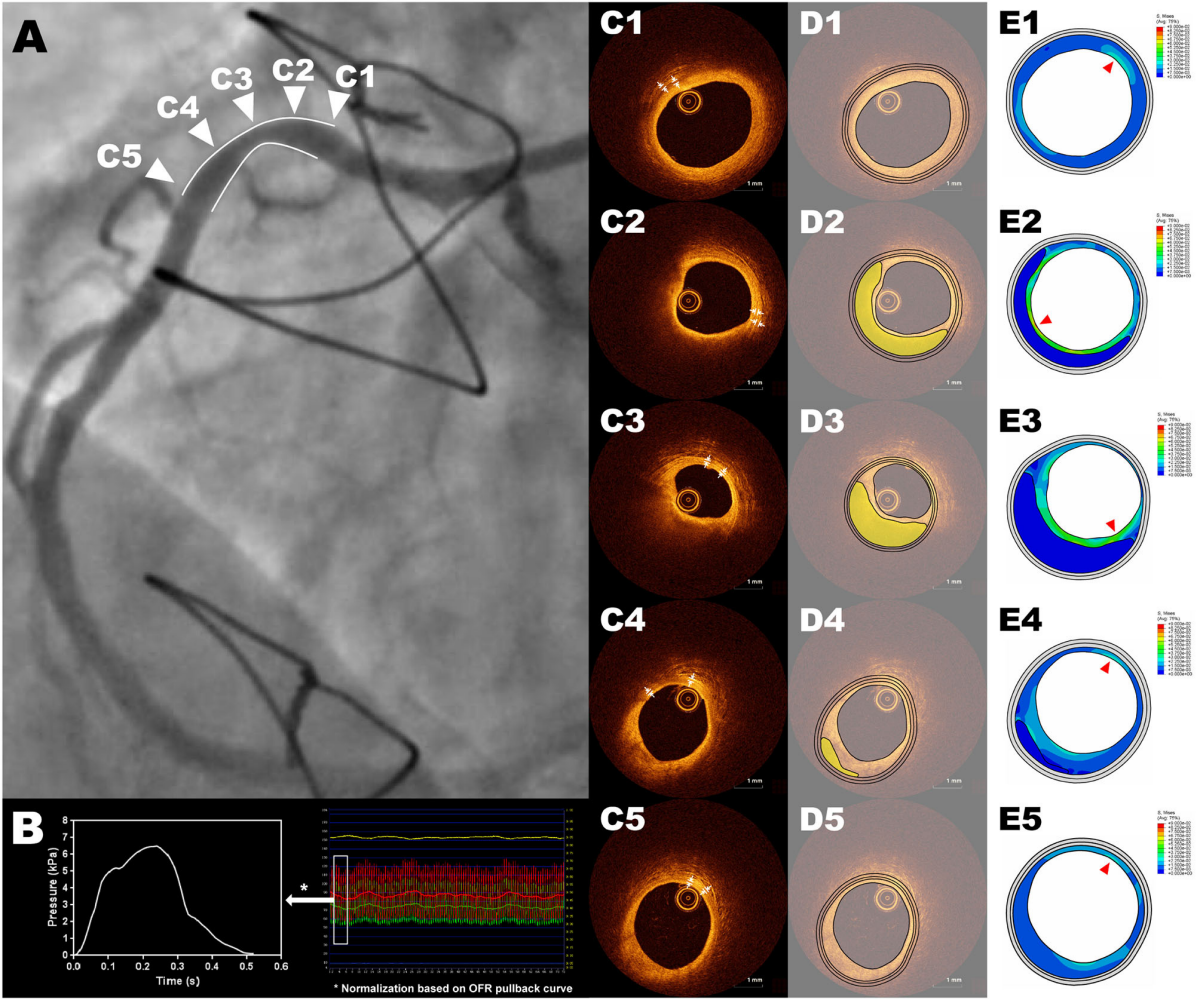
Representative example of ΔPSS analysis of an intermediate RCA lesion. Cross-sections **(C1-C5)** correspond to the five representative positions in the angiography showed in panel **(A)**, with **(C1)** as the cross-section of proximal reference, **(C2**) as the cross-section of proximal shoulder, **(C3)** as the cross-section of minimal lumen area, **(C4)** as the cross-section of distal shoulder and **(C5)** as the cross-section of distal reference. The 2-dimensional FEA model is loaded with the position-specific intracoronary pressure derived from intracoronary tracing data, by normalizing the computed OFR pullback curve between the resting aortic pressure tracing data and distal coronary pressure tracing data **(B)**. The three-layer geometric models were reconstructed based on an automatic plaque delineation algorithm (18) **(D1–D5)**, where the lipidic plaque is shown in yellow. The thicknesses of media and adventitia were manually measured from OCT cross-sections **(C1–C5)**. **(E1–E5)** show the stress distribution with the red triangles pointing the positions with largest ΔPSS. FEA, finite element analysis; RCA, right coronary artery; OCT, optical coherence tomography; OFR, optical flow ratio; ΔPSS, delta plaque structural stress.

Five OCT cross-sections were selected at representative positions for each lesion, whenever available (Figures 1C1–C5): 1) proximal and distal references (PR, DR): immediately adjacent cross-sections to the lesion, at the proximal and distal edges, respectively, where neither plaque nor remodeling were observed in OCT and angiography; 2) minimal lumen area (MLA): the cross-section with minimal lumen area in OCT; 3) proximal and distal shoulders (PS, DS): midpoints between PR and MLA or DR and MLA, respectively. In case the PR or DR are not present in certain lesion, the midpoint between proximal edge and MLA or distal edge and MLA will be used for PS or DS, respectively.

### Geometric Model Reconstruction

The lumen contours of selected OCT cross-sections were automatically delineated using OctPlus software (Pulse Medical Imaging Technology, Shanghai, China). The contour of the internal elastic lamina (IEL) was then identified or extrapolated from adjacent cross-sections, according to a previously validated method (18). The plaque composition was then automatically analyzed at every cross-section in the region encompassed between IEL and the lumen contour, using artificial intelligence (18). The mechanical-relevant plaque components considered for the current FEA in the intima were lipids, calcium and fibrous tissue. Media and adventitia were also incorporated into the geometric model by measuring their thickness on OCT and offsetting the IEL contour uniformly (Figures 1C1–C5,D1–D5). In case media and adventitia were not visible at the cross-section, their thickness on adjacent OCT cross-section was selected.

### Biomechanical Analysis

The simulation was performed with the commercially available FEA software ABAQUS (Version 6.13, Dassault Systemes Simulia Corp., Providence, RI, USA). Intimal lipidic plaque and calcific plaque were modeled as isotropic, hyperelastic materials as described by Mooney-Rivlin strain energy density function:

$$\begin{array}{l} W=C_{1}\left(I_{1}-3\right)+C_{2}\left(I_{2}-3\right)+D_{1}\left(\exp\left(D_{2}\left(I_{1}-3\right)\right)-1\right) \end{array}$$

where *I*_1_ and *I*_2_ are the first and second strain invariants. *C*_1_, *C*_2_, *D*_1_ and *D*_2_ are material constants adapted from previous studies (19, 20).

Intimal fibrous tissue, media and adventitia were modeled as anisotropic, hyperelastic materials, according to Holzapfel model:

$$\begin{array}{l} W=\mu \left(I_{1}-3\right)+\frac{k_{1}}{k_{2}}\left(\exp\left\{k_{2}\left[\left(1-\rho \right){\left(I_{1}-3\right)}^{2}+\rho {\left(I_{4}-1\right)}^{2}\right]\right\}-1\right) \end{array}$$

where μ, *k*_1_ and *k*_2_ are material constants adapted from previous studies (21–23).

Position-specific pressure condition derived from intracoronary tracing data was applied to the 2-dimensional (2D) FEA model for each cross-section. From OCT images the OFR pullback was firstly computed using a recently validated software package (OctPlus, Pulse medical imaging technology, Shanghai, China) (17, 24, 25). By normalizing the computed OFR pullback curve between the resting aortic pressure and distal coronary pressure tracing data, the position-specific intracoronary pressure at each OCT cross-section could be precisely estimated, also given the excellent agreement between OFR and FFR (17). The change in coronary pressure, i.e., the relative pressure, was then computed by subtracting the estimated diastolic pressure from the cyclic pressure (Figure 1B), which was used as the mechanical load for simulations. The 2D FEA models were then meshed with three-node or four-node linear, hybrid elements. Large deformation formulation and plane strain assumption were used for simulation. The rotational freedom was restricted to prevent the model from rolling while enabling its radial deformation. By submitting the FEA model to ABAQUS/Explicit Solver, the dynamic change of lumen and the plaque structural stress distribution at each cross-section during the cardiac cycle could then be simulated (Supplementary Video 1).

After the simulation, the maximal superficial von Mises stress on the vessel wall at maximal pressure load moment was denoted as OCT-derived change in plaque structural stress (ΔPSS). The thickness of the superficial layer depends on the size of the meshes in the FEA model which was <50 μm. Lumen diameter change (LDC) and percent lumen diameter change (LDC%) during cardiac contraction were used for presenting lumen deformation and relative lumen deformation. The LDC equals to the maximal lumen diameter minus minimal lumen diameter over the cardiac cycle. The LDC% is computed by dividing LDC with the minimal lumen diameter.

### Statistics

Descriptive statistics of continuous variables are reported as mean ± SD or median (quartiles) as appropriate, while those of categorical variables are presented as counts (percentages). The difference between groups was tested using independent sample *t*-test, Mann-Whitney test or One-way Analysis of Variance (ANOVA), as appropriate. Paired *t*-test, Wilcoxon signed-rank test or repeated measures ANOVA were used for pair-wise comparison, as appropriate. To evaluate the statistical differences of simulated lumen deformation and plaque structural stress at different locations of the lesion, the generalized estimation equation (GEE) analyses were performed. Statistical assessments were performed with MedCalc version 19.5.6 (MedCalc Software, Ostend, Belgium) and SPSS version 27.0.1.0 (SPSS Inc., Chicago, Illinois). A 2-sided value of *p* < 0.05 was considered to be statistically significant.

## Results

### Baseline Clinical and Lesion Characteristics

**Figure 2:**
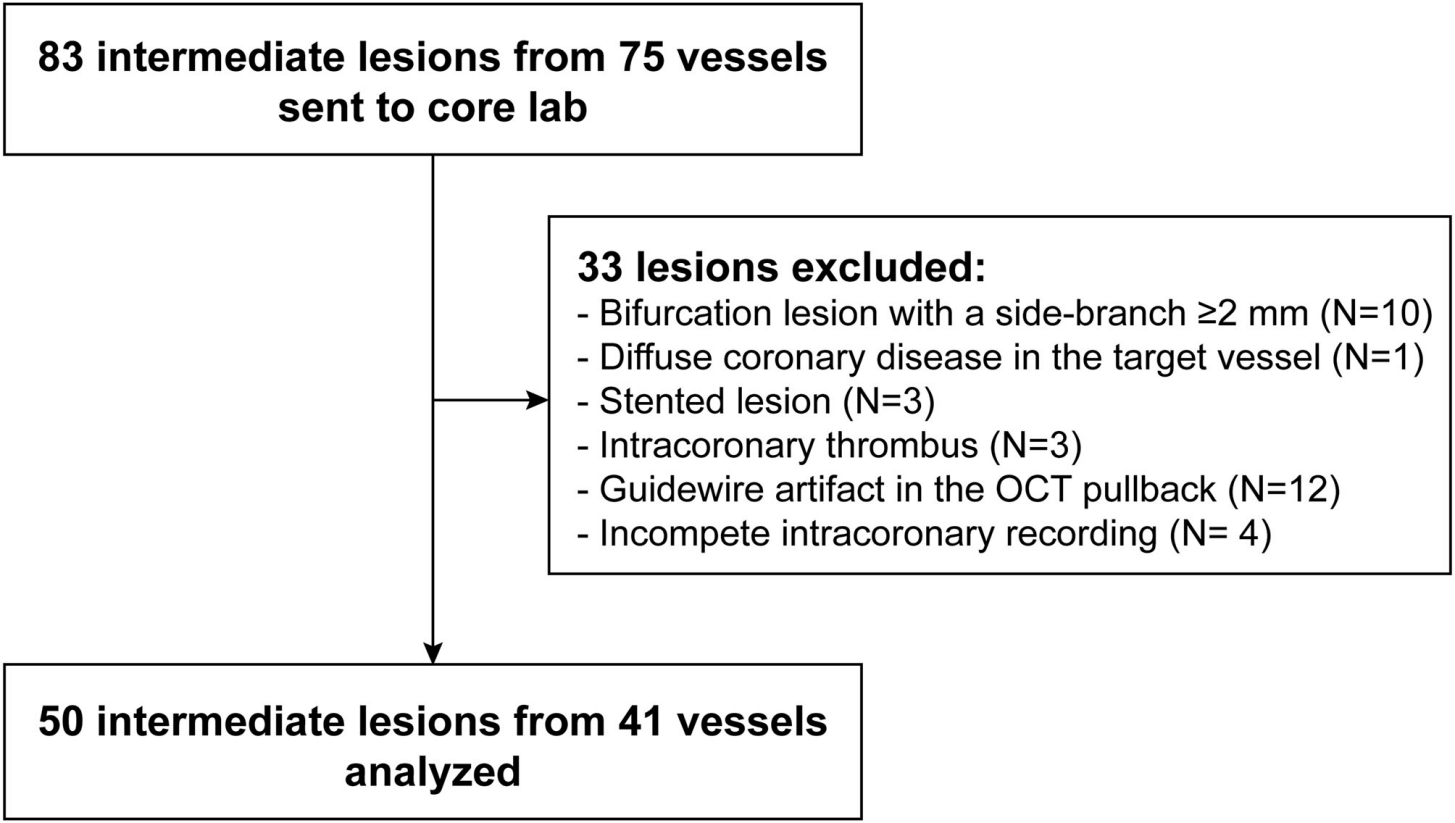
Study flow chart. FFR, fractional flow reserve; OCT, optical coherence tomography.

A total of 83 intermediate lesions from 75 vessels were enrolled. Thirty-three lesions were excluded from FEA due to bifurcation (*n* = 10), diffuse disease (*n* = 1), stented lesion (*n* = 3), intracoronary thrombus (*n* = 3), guidewire artifact (*n* = 12) or incomplete intracoronary recording (*n* = 4), resulting in 50 lesions suitable for biomechanical analysis (Figure 2).

Baseline demographic and lesion characteristics are presented in Tables 1, 2, respectively. The mean age of the patients was 63 ± 11 years. Half of the lesions (50.0%) were located at the proximal segment of the target vessel.

**Table 1:** Baseline demographic characteristics.

**Patients (*****N*** **= 37)**	
Age, years	63 ± 11
Women	3 (8.1%)
BMI, kg/m^2^	28.4 [25.4, 29.9]
Diabetes mellitus	12 (32.4%)
Hypertension	25 (67.6%)
Hyperlipidemia	17 (46.0%)
Current smoker	12 (32.4%)
Family history of CAD	3 (8.1%)
Previous PCI	29 (78.4%)
Previous CABG	1 (2.7%)
Previous MI	21 (56.8%)
**Clinical presentation**	
Stable Coronary Heart Disease	29 (78.4%)
Unstable Angina	4 (10.8%)
NSTEMI	4 (10.8%)

*Data are presented as mean ± SD, median (quartiles), or n (%), as appropriate*.

*BMI, body mass index; CAD, coronary artery disease; CABG, coronary artery bypass surgery; NSTEMI, Non-ST-elevation myocardial infarction; MI, myocardial infarction; PCI, percutaneous coronary intervention*.

**Table 2:** Baseline lesion characteristics.

**Vessels (*****N*** **= 41)**	
**Interrogated vessel**	
Left anterior descending	23 (56.1%)
Diagonal	4 (9.8%)
Left circumflex	0 (0.0%)
Obtuse marginal	2 (4.9%)
Ramus intermedius	1 (2.4)
Right coronary artery	11 (26.8%)
**Lesions (*****N*** **= 50)**	
**Lesion location**	
Proximal segment	25 (50.0%)
Middle segment	19 (38.0%)
Distal segment	6 (12.0%)
Minimal Lumen Area, mm^2^	2.70 [2.16, 3.11]

*Data are presented as mean ± SD, median (quartiles), or n (%), as appropriate*.

### Plaque Morphology and Composition

Quantification of the OCT images was performed using the OctPlus software package (Pulse medical imaging technology, Shanghai, China). Automatic plaque characterization and delineation of the IEL from OCT images by the software was performed using artificial intelligence algorithm and recently validated with high accuracy (18). Average lumen diameter, plaque burden and fibrous cap thickness in the representative positions are presented in Table 3. MLA had the smallest lumen diameter (1.82 [1.60, 1.97] mm) and the largest plaque burden (69.11 ± 9.11%) of all the representative positions. Fibrous cap thickness was numerically smaller in PS than in MLA and DS, but it did not reach statistical significance (PS vs. MLA vs. DS: 154.1 [60.8, 242.2] μm vs. 168.9 [54.2, 264.7] μm vs. 212.4 [92.7, 253.2] μm, *p* = 0.48). The plaque composition and microfeatures of the lesions included in current study are presented in Table 4. Forty-one lesions (82.0%) have more than one plaque phenotypes. By using the predominant plaque phenotype for each lesion, a total of 28 fibroatheromas, 8 fibrotic plaques and 14 fibrocalcific plaques were included in the present study.

**Table 3:** Plaque morphologies.

**Position within the lesion**	**Lumen diameter (mm)**	**Plaque burden (%)**	**Cap thickness (μm)**
PR (*N* = 10)	3.94 [2.92, 4.21]	32.47 ± 5.78	–
PS (*N* = 50)	2.46 [2.13, 2.95]	56.91 ± 5.67	154.1 [60.8, 242.2]^*^
MLA (*N* = 50)	1.82 [1.60, 1.97]	69.11 ± 9.11	168.9 [54.2, 264.7]^*^
DS (*N* = 50)	2.40 [2.15, 2.63]	53.82 ± 11.57	212.4 [92.7, 253.2]^*^
DR (*N* = 17)	2.91 [2.74, 3.18]	32.36 ± 8.70	–

* *Cap thickness was not recorded in 3 PS, 2 MLA and 14 DS because only fibrous plaque was observed*.

*PR, proximal reference; PS, proximal shoulder; MLA, minimal lumen area; DS, distal shoulder; DR, distal reference*.

**Table 4:** Plaque composition and microfeatures.

**Position within the lesion**	**Number of lipidic plaques**	**Mean lipidic plaque area (mm^**2**^)**	**Number of calcific plaques**	**Mean calcific plaque area (mm^**2**^)**
PS (*N* = 50)	49	2.18 [1.36, 3.22]	29	0.65 [0.39, 1.83]
MLA (*N* = 50)	51	2.28 [1.24, 3.85]	27	0.70 [0.31, 0.85]
DS (*N* = 50)	38	1.41 [0.87, 2.91]	22	0.48 [0.22, 0.89]

*PS, proximal shoulder; MLA, minimal lumen area; DS, distal shoulder*.

Among all 177 interrogated cross-sections, more than half of them (92 cross-sections) have a good (external elastic lamina circumference ≥270°) or moderate visibility (270° > external elastic lamina circumference ≥ 180°) of the media (54 and 38 cross-sections, respectively). While the media were completely invisible in 14 (7.9%) cross-sections, where the artificial intelligence algorithm used their adjacent cross-sections for IEL delineation. In these cases, the mean number of skipped cross-sections for manual media and adventitia thicknesses measurement was 5.6 ± 6.8 frames.

### Lumen Deformation and Plaque Structural Stress

**Figure 3:**
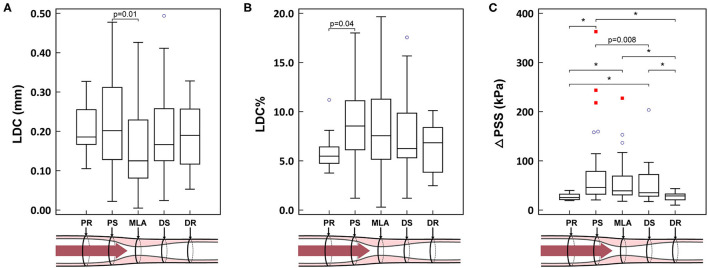
**(A–C)** Biomechanical results in different locations of the lesion. *p < 0.001. PR, proximal reference; PS, proximal shoulder; MLA, minimal lumen area; DS, distal shoulder; DR, distal reference; LDC, lumen diameter change; LDC%, percent lumen diameter change; ΔPSS, delta plaque structural stress. The blue circles represent the mild outliers, and the red squares represent the extreme outliers.

A representative example of ΔPSS analysis is shown in Figure 1 and Supplementary Video 1. The LDC, LDC% and ΔPSS across the lesion are presented in Table 5 and Figure 3. Absolute LDC was smaller at MLA than at both reference cross-sections (PR and DR), but LDC% was larger. Positive correlation was observed between LDC and lumen diameter (*r* = 0.53, *p* < 0.01). However, no correlation was observed between LDC% and lumen diameter.

**Table 5:** Biomechanical results.

**Position within the lesion**	**LDC (mm)**	**LDC%**	**ΔPSS (kPa)**
PR (*N* = 10)	0.19 [0.17, 0.26]	5.48 [4.75, 6.43]	25.2 [21.2, 32.3]
PS (*N* = 50)	0.20 [0.13, 0.31]	8.55 [6.13, 11.12]	45.7 [32.3, 78.6]
MLA (*N* = 50)	0.13 [0.08, 0.23]	7.56 [5.17, 11.28]	39.0 [30.8, 69.1]
DS (*N* = 50)	0.17 [0.13, 0.26]	6.26 [5.31, 9.85]	35.1 [28.2, 72.3]
DR (*N* = 17)	0.19 [0.12, 0.26]	6.84 [3.85, 8.39]	28.6 [20.8, 32.6]

*PR, proximal reference; PS, proximal shoulder; MLA, minimal lumen area; DS, distal shoulder; DR, distal reference; LDC, lumen diameter change; ΔPSS, delta plaque structural stress*.

ΔPSS was significantly smaller at the reference cross-sections than at MLA or at the shoulders (PR vs. PS, MLA and DS: 25.2 [21.2, 32.3] kPa vs. 45.7 [32.3, 78.6] kPa, 39.0 [30.8, 69.1] kPa and 35.1 [28.2, 72.3] kPa; *p* < 0.001, *p* < 0.001 and *p* < 0.001, respectively). In paired analysis per lesion, a significant ΔPSS gradient was observed across the plaque (PS-MLA-DS, *p* = 0.046; test for linear trend: *p* = 0.012). ΔPSS at PS was significantly larger than at DS (*p* = 0.02), while ΔPSS at MLA tended to have intermediate values between PS and DS (Figure 3). Within all 49 lesions with lipidic plaques, the highest ΔPSS occurred at the cross-sections with thinnest cap in 49.0% (24) of these lesions. While for 22 lesions (44.9%), the highest ΔPSS occurred at the cross-sections with lipidic plaques but thicker cap thickness. Three (6.1%) lesions were observed to have the highest ΔPSS at cross-sections without any lipidic plaque.

### Normal vs. Diseased Segments

**Figure 4:**
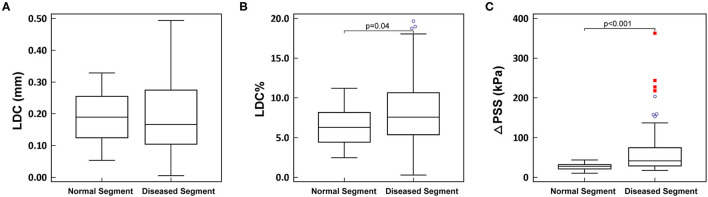
**(A–C)** Comparison between normal segment and diseased segment. LDC, lumen diameter change; LDC%, percent lumen diameter change; ΔPSS, delta plaque structural stress. The blue circles represent the mild outliers, and the red boxes represent the extreme outliers.

The analyzed OCT cross-sections were divided into two groups: 1) normal segments, comprising PR and DR; 2) diseased segments, comprising PS, MLA and DS. LDC was numerically smaller in diseased segments compared to normal segments, but statistically non-significant (0.17 [0.10, 0.27] mm vs. 0.19 [0.13, 0.25] mm, *p* = 0.69). While both LDC% and ΔPSS were significantly larger in diseased segments than in normal segments (LDC%: 7.57 [5.38, 10.64]% vs. 6.29 [4.44, 8.17]%, *p* = 0.002; ΔPSS: 41.8 [29.1, 74.6] kPa vs. 27.7 [21.2, 32.1] kPa, *p* < 0.001) (Figure 4).

In normal segments, a good correlation was observed between maximal pressure load and ΔPSS and (*r* = 0.72, *p* < 0.001). Nonetheless, this correlation was significantly worse in diseased segments (*r* = 0.42, *p* < 0.001; difference *p* = 0.04), wherein relatively low pressure loads often resulted in high ΔPSS.

### Correlation of Stress Parameters With Morphological Features of Plaque Vulnerability

ΔPSS was positively correlated with plaque burden (*r* = 0.37, *p* < 0.001) while negatively correlated with fibrous cap thickness (*r* = −0.25, *p* < 0.001). Good correlation was observed between ΔPSS and LDC% (*r* = 0.78, *p* < 0.001).

Moderate correlation was observed between ΔPSS and lipidic plaque area (LPA) (*r* = 0.44, *p* < 0.001), while no significant correlation was observed between ΔPSS and calcific plaque area. LPA showed good correlation with both lumen deformation and relative lumen deformation (LPA-LDC: *r* = 0.55, *p* < 0.001; LPA-LDC%: *r* = 0.88, *p* < 0.001).

### Computational Performance of ΔPSS Assessment

Using an off-the-shelf workstation with a quadcore Intel i7-4790 processor (Intel Corporation, Santa Clara, CA; 3.6 GHz) and 8GB of RAM, the average simulation time for analysis of each FEA model was 12.5 ± 10.2 min, and the estimated time for the whole FEA analysis process was <30 min.

## Discussion

In this study, we present for the first time a new methodology to calculate the changes in plaque structural stress within the cardiac cycle *in vivo* using OCT images and FEA simulation. The changes of superficial plaque structural stress on vessel wall are the main focus of our current study since it tends to be closely related to plaque rupture and subsequent acute coronary events (26–29).

The key findings of this study can be summarized as follows: 1) ΔPSS provides a feasible and reasonable approach for OCT-based biomechanical assessment; 2) diseased segments, especially the proximal shoulder and minimal lumen area of the lesion, bear the highest ΔPSS, thus highlighting the critical importance of these sites for future biomechanical studies of plaque vulnerability and prediction of event risk; 3) correlation between ΔPSS and plaque morphology is consistent with previous clinical and imaging studies, thus reassuring the rationale of our method.

This novel method is original in many aspects, including the automatic plaque characterization from OCT images using artificial intelligence, the incorporation of the three-layered structure of the vessel wall into 2D FEA model, the accurate position-specific load derived from intracoronary pressure tracing data. Previous studies performed coronary plaque stress simulation (27), but most of them were based on coronary angiography or intravascular ultrasound (IVUS), with inherent limitations in plaque characterization and inferior image resolution, as compared with OCT. Additionally, rigid-body assumption restricting all deformation was applied to most FEA models hitherto (20, 30, 31).

The first advantage of the current approach is the higher image resolution provided by OCT, enabling the detailed description of coronary lumen, plaque morphology and composition which are essential for FEA analysis. The significant lower correlation between load and ΔPSS in diseased segments than in normal segments (*r* = 0.42 vs. 0.72, *p* < 0.001) strongly suggests a crucial role of plaque morphology and composition in determining the actual plaque stress. Chau et al. have also proposed an FEA method to derive plaque stress from OCT images (32). However, a fixed pressure from 0 to 120 mmHg was applied to all models, which might not be realistic since the geometry of the vessel was not imaged under zero-pressure condition. In addition, the same isotropic material properties were applied to the whole artery wall, ignoring the anisotropy of media and adventitia and the mechanical differences between them. Although OCT has limited tissue penetration, as compared with IVUS, and notwithstanding the high attenuation of near-infrared waves in lipids, apparently precluding the imaging of media and adventitia in diseased segments (16), recent studies have proven that the external elastic lamina can be identified for ≥180° in most of the OCT cross-sections (33). Thus, the media contour could be extrapolated considering its circular or elliptical geometry. For OCT cross-sections with invisible IEL, which only accounts for 7.9% of the total cross-sections in this study, our artificial intelligence algorithm will refer to adjacent OCT cross-sections and estimate the contour of IEL. This principle was externally validated for the same specific software used in the current study, providing high diagnostic accuracy for delineation of the media contour and tissue characterization within the plaque, including lipids (90.5%) (18). In this study, we mainly focused on the superficial cyclic plaque structural stress on vessel wall, which is more likely to be affected by the different plaque components in near-lumen regions, as compared to outer layers as media and adventitia. During the analysis, we also observed that the slight deviation, i.e., thickness and contour, in media and adventitia have a relatively small impact on simulation results. While for superficial layers, even a tiny change, e.g., lumen contour and fibrous cap thickness, will cause great difference in ΔPSS. It has also been proven by previous studies that the most crucial impact on stress computation comes from the superficial wall adjacent to lumen, and that the vascular structure determined by OCT provides adequate basis for biomechanical analysis (23, 32). Nevertheless, when focusing on the stress distribution at deeper layer, e.g., behind the plaque or near the outer vessel boundary, more precise simulation results might be achieved by incorporating more accurate delineation of media and adventitia, especially for lesions with large lipidic plaque burden where the outer boundary is invisible due to light attenuation in OCT. In such cases, IVUS with higher tissue penetration could serve as a complemental modality (34). By combining detailed evaluation of plaque morphology provided by OCT and assessment of the entire vessel structure provided by IVUS, a more precise geometric model for FEA should become obtainable, leading to a more accurate biomechanical assessment especially for outer layers (35). In the future, with the development of automatic OCT-IVUS co-registration algorithm and/or hybrid intravascular imaging system with combined OCT and IVUS probes (36), the accuracy of PSS assessment could be further improved with great efficiency.

A complete vessel architecture with three-layer structure was applied for simulation in this study. Very few studies have incorporated the three-layered structure of the vessel into the FEA simulation hitherto. This might be a potential limitation of previous studies because stress distribution heavily depends on the physical properties of the material and the layered arrangement of the vessel wall (21, 37–39). The different composition and stiffness of intima, media and adventitia determine their different roles during loading. The stiffness of intima widely varies depending on the different plaque components. Conversely, media and adventitia use to have more predictable mechanical properties: the structured arrangement of smooth muscle cells in the media confers this layer high resistance to load, while the helically arranged wavy collagen fibrils and elastic fibers in adventitia make it more compliant to pressure than media under normal load (40, 41).

Accurate model load using intracoronary pressure registration has been instrumental to render precise stress estimations. The model, however, was only loaded with the change in coronary pressure, instead of using the whole pressure recording, in order to simplify and expedite the calculation. The stress change over the cardiac cycle in atherosclerotic plaques has been proven to correlate with the incidence of major adverse cardiovascular events (42) and the general relation between plaque morphology and plaque stress remains intact irrespective of the inclusion or exclusion of the initial stress in the model (43). On this rationale, the decision of using only pressure change seems justified. Loading the model with the whole pressure recording might have added marginal accuracy to the estimations, but at the price of exponentially increasing the complexity of the analysis and subsequently the time required for it (43–45). For models to be loaded with the whole pressure recording, accurate computation of zero-pressure state is one of the prerequisites. Several methods have been proposed for the estimation of initial stress. A preshrinkage algorithm by Huang et al. iteratively shrinks the *in-vivo* plaque geometry before the whole pressure loading to estimate the zero-pressure state and initial stress (44, 45). However, manual adaptation was required in each iteration to compare the computed geometry with the image-derived real geometry and adjust the geometry for the next iteration, which might be too complicated for daily practice. Speelman et al. proposed a Backward Incremental method that requires no manual input (43). However, the vessel geometry under certain fixed intracoronary pressure is one of the prerequisites for the intimal stress estimation, making this method not suitable for *in vivo* assessment. Theoretically, zero-pressure state and initial stress could be accurately estimated from the geometrical models of the same cross-section at both diastole and systole, where the imaging catheter is required to be fixed at the same position for at least one cardiac cycle. However, this kind of relative stillness between catheter and vessel is technically difficult to achieve, considering the impact of cardiac motion and vessel contraction (46). Thus, the gain in incorporating whole pressure recording might be partly canceled by the relative displacement between the imaging catheter and the analyzed cross-section during cardiac cycle.

The consistency of the plaque stress calculated by this novel method with previous clinical findings and biomechanical studies is reassuring of the validity of our approach. Autopsy studies have linked high plaque stress with plaque rupture (13, 47). Likewise, imaging studies have associated high plaque stress with acute coronary syndromes (14). This evidence supports the biomechanical hypothesis for plaque rupture: the repetitive cyclic stress would end up breaking the plaque, like repetitively bending a paper leads to its weakening and fracture (1). In line with this evidence, normal coronary segments bear significantly lower ΔPSS than diseased segments in our study. Moreover, it is known that strain and plaque stress are higher upstream than downstream in the atherosclerotic plaque (48, 49), i.e., higher in the proximal segments than in the distal segments of the plaque, and that most plaque ruptures occur in these proximal segments of the lesion (30, 48, 50, 51). Our results, finding a plaque stress gradient from the PS to the MLA and ultimately to the DS, are in line with this preceding evidence. In addition, ΔPSS showed positive correlation with LPA and negative correlation with fibrous cap thickness, thus confirming a direct association between ΔPSS and morphologically rupture-prone plaque, i.e., TCFA. Nonetheless, both correlations were relatively weak (*r* = 0.37 and−0.25, respectively) and only around half (49.0%) of the lesions with lipidic plaques have the highest ΔPSS located at the thinnest cap sites, thus suggesting a mismatch between the histological and biomechanical evaluation of vulnerability. This observation may at least partially account for the limited prognostic value of TCFA alone (9–12).

In this study, the mean LDC% in normal segment is 6.29%, slightly smaller than the range of 7–14% observed by several previous studies from groups of healthy people aged from 8 to 60 (52–54). Since the coronary artery stiffens with age and disease, the relatively smaller LDC% of 6.29% in this study seems reasonable considering that our material property for intimal fibrous tissue was adopted from patients with an average age of 66 (21). The LDC is larger in normal segments compared to the diseased segments, though statistically non-significant. This finding is in line with previous IVUS studies that normal segments are more compliant to deformation (55). Conversely, significantly larger LDC% in diseased segments were observed in the present study (7.57% vs. 6.29%, *p* = 0.04). A possible explanation for this phenomenon is that 82% of the diseased segments have lipidic plaques, which are softer and thus tend to have larger relative deformation. Good correlation between LPA and %LDC further elucidates this point (*r* = 0.88, *p* < 0.001).

### Clinical Perspectives

TCFA is currently recognized as a precursor for plaque rupture. However, several issues regarding image-based TCFA detection remain unsolved, including modest interobserver agreement and inconsistent definitions between studies (56). In addition, most TCFAs do not cause symptomatic rupture (9–11), revealing the fact that histological assessment alone is not enough for ACS prevention.

Plaque stress evaluation based on FEA might serve as a supplementary strategy. The location of peak cyclic plaque stress might be helpful to predict the risk of rupture and hence for ACS prevention. However, the length of a focal lesion is 12–30 mm, with 60–150 OCT cross-sections at the highest pullback speed (57, 58). Therefore, plaque stress evaluation would be very time-consuming if the whole lesion were scanned. For future studies, limiting the analysis to high-risk locations, especially for lipid rich plaques which are at higher propensity to rupture, might be instrumental for fast and efficient risk stratification.

Interestingly, LDC% showed good correlation with both ΔPSS (*r* = 0.78, *p* < 0.001) and LPA (*r* = 0.88, *p* < 0.001) in our study. These findings invite us to explore the possibility of using angiography-derived lumen deformation for the estimation of lipidic burden and for simplified identification of high-risk sites for rupture. Considering the relatively short modeling and simulation time of ΔPSS, the hereby described method might also be integrated into the current OCT-based FFR computation system, where the morphological, histological and biomechanical assessments could be achieved efficiently within one single OCT pullback.

### Limitations

The current study is limited by its *post-hoc* design and its relatively small sample size. Nevertheless, all intermediate lesions with both OCT and intracoronary pressure tracing were enrolled, following strictly predefined inclusion/exclusion criteria, thus minimizing the selection bias. Besides, not all interrogated lesions had PR and DR.

In this study, the FEA models were reconstructed in 2D without incorporating the shear stress and mechanical forces in the axial direction (59–61). The homogenous material property within lipidic and calcific plaque and the uniform assumption for media and adventitia might also introduce error into the simulation. Besides, the effect of residual stress was not considered since it was currently immeasurable. The lumen deformation might also be different from real situation depending on the prevailing diastolic blood pressure levels. In addition, for cross-sections with invisible IEL, the vessel boundaries estimated by the artificial intelligence might be less accurate and lead to imprecise simulation results. However, the impact of this limitation on superficial ΔPSS is negligible. Although the simulation results are in line with clinical findings, due to the complexity of our current approach combining OCT, FFR and FEA model together, there is no single “gold standard” for validation. Besides, only presentative positions were analyzed for each lesion in this feasibility study. We did not perform frame-by-frame analysis considering that the reliability in tissue characterization might be impaired for cross-sections with side branches. Future prospective studies with larger sample sizes are warranted to investigate the prognostic value and the clinical usefulness of ΔPSS and other ΔPSS-related parameters.

The current approach, using intracoronary pressure recordings, is exquisitely accurate, but it makes the method complex and expensive to routine clinical implementation. In addition, only OCT images without guidewire artifact were analyzed in this pilot study. Future simplifications of this approach might facilitate the applicability of this assessment in the cathlab, provided they rendered acceptable accuracy.

## Conclusions

Plaque structural stress over the cardiac cycle can be estimated from OCT images, using automatic plaque characterization and FEA, on a feasible fully automated process aided by artificial intelligence. The results of this novel approach are consistent with previous clinical and biomechanical studies, showing higher plaque stress in diseased vs. normal segments. The highest stress at the proximal shoulder and MLA indicates the critical rupture-prone sites for efficient biomechanical assessment in the future.

## Data Availability Statement

The dataset can be shared upon reasonable request. Requests to access these datasets should be directed to the corresponding author.

## Ethics Statement

The studies involving human participants were reviewed and approved by Local Ethic Committee of Campo de Gibraltar Health Trust. The patients/participants provided their written informed consent to participate in this study.

## Author Contributions

JH: analysis of the data and draft of the manuscript. WW and ST: concept design and draft of the manuscript. FY, TX, RL, JW, YL, and XL: analysis of the data. JG-C, PS, YO, LW, and DT: crucial revision of the manuscript. All authors contributed to the article and approved the submitted version.

## Funding

This work was supported in part by grants from the National Natural Science Foundation of China [grant numbers 82020108015 and 81871460] to ST and [grant number 11972117] to LW, and by the Science Foundation Ireland Research Professorship grant RSF 1413 to WW, and by grants from the Science and Technology Committee of Shanghai Municipality (No. 19411963200) to YL.

## Conflict of Interest

WW reports research grants and honoraria from MicroPort, medical advisor of Rede Optimus Research and co-founder of Argonauts, an innovation facilitator. ST reports research grants and consultancy from Pulse medical imaging technology. PS reports personal fees from Sino Medical Science Technology, Philips/Volcano, Xeltis, HeartFlow, and SMT, outside the submitted work. The remaining authors declare that the research was conducted in the absence of any commercial or financial relationships that could be construed as a potential conflict of interest.

## Publisher's Note

All claims expressed in this article are solely those of the authors and do not necessarily represent those of their affiliated organizations, or those of the publisher, the editors and the reviewers. Any product that may be evaluated in this article, or claim that may be made by its manufacturer, is not guaranteed or endorsed by the publisher.
