# Supplementary material for: Optical Coherence Tomography-Derived Changes in Plaque Structural Stress Over the Cardiac Cycle: A New Method for Plaque Biomechanical Assessment
Source: Front Cardiovasc Med. 2021 Nov 4;8:715995. doi: 10.3389/fcvm.2021.715995 (PMC8600113; doi:10.3389/fcvm.2021.715995)
Supplement: Supplementary file 2 [file Image_1.PDF]

Supplementary Material

1 Supplementary Figures and Tables

1.1 Supplementary Figures

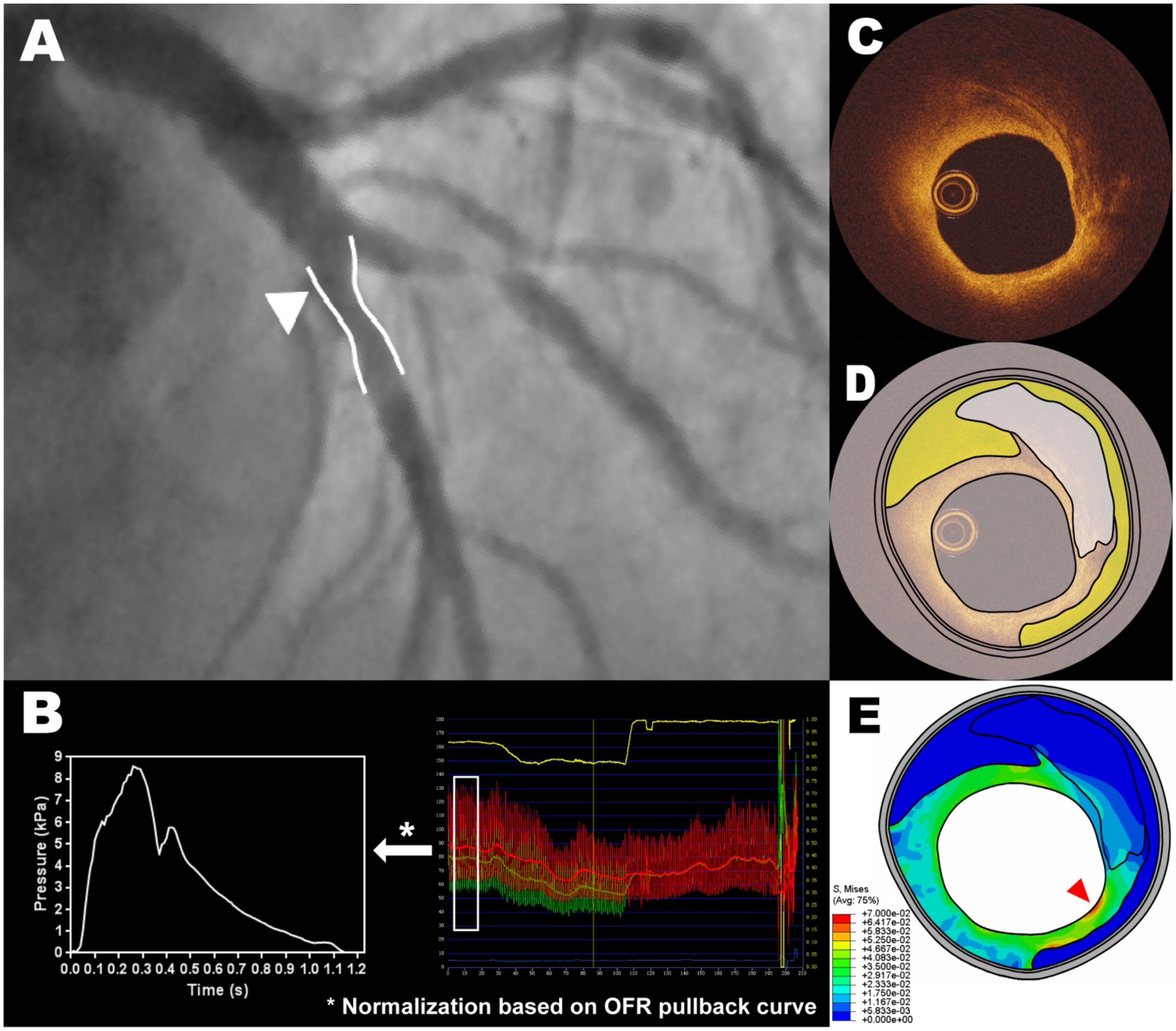

Supplementary Figure 1. C and E correspond to the proximal shoulder site of the LAD lesion indicated by the white arrow on the angiography shown in panel A. The 2-dimensional FEA model is loaded with the position-specific intracoronary pressure computed from intracoronary tracing data, by normalizing the computed OFR pullback curve between the resting aortic pressure tracing data and distal coronary pressure tracing data (B). The three-layer geometric models were reconstructed based on an externally validated automatic plaque delineation algorithm (D), where the lipidic plaque is shown in yellow and calcific plaque is shown in white. The thicknesses of media and adventitia were

manually measured from adjacent OCT cross-sections. E shows the stress distribution with the red triangle pointing toward the position with largest ΔPSS.

FEA = finite element analysis; LAD = left anterior descending artery; OCT = optical coherence tomography; OFR = optical flow ratio; ΔPSS = delta plaque structural stress.
